# Meta^QM^: Exploring the Role of QM Calculations in Drug Metabolism Prediction

**DOI:** 10.3390/ijms262412087

**Published:** 2025-12-16

**Authors:** Alessio Macorano, Serena Vittorio, Angelica Mazzolari, Alessandro Pedretti, Giulio Vistoli

**Affiliations:** Dipartimento di Scienze Farmaceutiche, Università degli Studi di Milano, Via Mangiagalli 25, 20133 Milan, Italyserena.vittorio@unimi.it (S.V.);

**Keywords:** metabolism prediction, MetaQSAR, random forest, metabolic database, site of metabolism

## Abstract

Understanding and predicting the metabolic fate of xenobiotics is essential in early drug discovery stages, as poor ADMET properties are a leading cause of new drug candidates’ failure. In silico metabolism modeling offers a way to design safer and more effective compounds. We present Meta^QM^, a set of random forest classifiers enhanced with quantum chemical descriptors to predict (i) the occurrence of metabolic reactions (Metaclass^QM^) and (ii) the site of metabolism (Metaspot^QM^). Models were trained on the MetaQSAR database, which contains 3788 expert-curated reactions divided into 3 main categories, 21 classes, and 101 subclasses. The descriptors used to train the models included physicochemical, constitutional, and stereo-electronic features computed at two levels of theory: PM7 (MOPAC 2016) and DFT (B3LYP/6-31G(d)). For Metaclass^QM^, the use of DFT descriptors led to improved classification performances by 10% at the class level and 8.6% at the subclass level, compared to PM7 descriptors. In Metaspot^QM^, both descriptor sets showed similar performance in SoM prediction across different datasets. DFT descriptors enhance the classification of metabolic reactions, while simpler methods suffice for the prediction of metabolic sites. These findings support the use of quantum descriptors in metabolism modeling workflows, balancing accuracy and computational cost.

## 1. Introduction

Metabolism prediction is gaining increasing relevance in recent years due to its capacity to prioritize molecules based on their metabolic stability and toxicity concerns [1]. When combined with other ADME predictive tools, in silico approaches allow a detailed pharmacokinetic profiling of novel compounds. Due to their affordability, computational approaches can be easily applied even to huge molecular libraries, thus anticipating the ADME/Tox profiling at early phases of drug discovery projects [2]. In the last few years, metabolism prediction has benefited from an increasing availability of biological data as well as from the endless progress in artificial intelligence algorithms, which allow patterns and relations to be unveiled even within complex data [3].

In the past, metabolism prediction studies were primarily focused on CYP-catalysed redox reactions by using both ligand and structure-based methods [4]. This can be mostly explained by considering both the relevance of these biotransformations and the lack of extended datasets for the other metabolic reactions. As mentioned above, the increased availability of metabolic data allowed the portfolio of predicted biotransformations to be markedly extended, including redox, hydrolysis, and conjugation reactions. The availability of ever more comprehensive datasets also enables the development of global methods [5] which try to predict the entire metabolic fate for an input molecule.

Among the available metabolic resources, we recently proposed a database, MetaQSAR [6], which shows two relevant advantages: (1) it derives from a manually curated meta-analysis of specialized literature and (2) it includes only metabolic reactions on xenobiotics, excluding reactions involving endogenous compounds or other secondary metabolic pathways. Moreover, it implements a very fine classification which subdivides all metabolic reactions into 3 major classes, 21 classes, and 101 subclasses, thus allowing a precise clustering of all collected data. These aspects render MetaQSAR an invaluable source of specific and highly accurate datasets for developing predictive models for metabolism assessment. MetaQSAR reliability was confirmed by several predictive studies involving both specific metabolic reactions and global approaches. Among them, MetaClass [7] and MetaSpot [8] are two tools based on MetaQSAR-derived datasets and aimed to predict the occurrence of specific metabolic reactions and the corresponding sites of metabolism (SoM), respectively.

The models implemented by the MetaClass and MetaSpot projects were based on an extended set of physicochemical, structural, and stereo-electronic descriptors computed by VEGA [9] and MOPAC tools and were developed by the Random Forest (RF) algorithm [10]. The feature importance analysis revealed that the stereo-electronic descriptors often play a primary role in describing the intrinsic reactivity of each molecule. The major role of these descriptors agrees with many previous studies, which emphasized the efficacy of reactivity descriptors to predict the regioselectivity of various metabolic reactions [11,12,13].

Notably, most of the previous studies involved stereo-electronic parameters as computed by semi-empirical calculations to reduce computational costs. As recently reviewed by Kostal [14], the exploitation of QM calculations for metabolism and toxicity prediction is still rare, even in industrial contexts, especially if compared to the use of QM simulations to rationalize the reactivity of covalent binders [15]. To allow the screening of large datasets, metabolism prediction has almost always involved simplified models based on knowledge-based rules or easy to calculate descriptors and fingerprints [16,17,18]. Nevertheless, the computational power routinely available for scientific projects should allow QM calculations to be applied to (at least) medium-size datasets of compounds, thus rendering these calculations amenable for the metabolism prediction task. Remarkably, QM calculations can provide a wide variety of stereo-electronic descriptors [19], which can be exploited (along with other descriptors) to develop enhanced predictive models by employing the rich arsenal of AI-based algorithms.

On these grounds, we performed an exhaustive campaign of QM calculations in which all the 2054 first generation substrates, already utilized by the MetaClass/MetaSpot study, underwent DFT-based full optimization and frequency calculations. The so-derived DFT-optimized structures, along with the computed stereo-electronic descriptors and resulting force-field parameters, have already been described [20] and freely released to the scientific community.

Hence, the primary objective of this study is the exploitation of the DFT-based stereo-electronic descriptors to develop classification models to predict: the occurrence of metabolic reactions (MetaClass^QM^), as well as the corresponding site of metabolism (SoM) (Metaspot^QM^). Stated differently, the study aims to conceptually repeat the predictive analyses performed by the MetaClass/MetaSpot studies, including the DFT-based stereo-electronic descriptors in lieu of those derived by semiempirical approaches. The comparison of the predictive performances should reveal the beneficial role of these DFT-based parameters.

To render the predictions as challenging and unbiased as possible, MetaClass^QM^ and Metaspot^QM^ involve balanced datasets, where the non-substrates possess the same reactive groups as the substrates. Furthermore, the predictive models were generated by including either stereoelectronic descriptors derived from semi-empirical calculations or those computed from DFT simulations. These two sets of models enable the precise evaluation of the role of QM calculations in each predicted metabolic reaction.

## 2. Results

### 2.1. MetaQSAR-Based Datasets

The predictive analysis reported here is based on metabolic reactions collected in the MetaQSAR database, focusing on first-generation reactions. The study involved a total of 3788 metabolic reactions, which were subdivided into 3 major classes (redox, hydrolysis, and conjugation), 21 classes, and 101 subclasses. Among them, predictive models were generated for 17 classes (Table S1) and 23 subclasses (Table S2) as they include datasets with at least 50 instances. In all studies, the datasets were generated by considering, for each metabolic reaction, all the molecules annotated as substrate (class S) as the positive class, while all remaining compounds were considered as non-substrates (class NS). This classification method poses the problem of false negatives, as a lack of experimental evidence regarding a given metabolic reaction does not necessarily imply that the resulting metabolite cannot occur. Indeed, a metabolic reaction may be unreported because the corresponding metabolites were undetectable using the adopted analytical methods or because they were not investigated, as the analyzed paper was designed with different objectives. As described in Section 4, the models were built using the Random Forest algorithm on the balanced dataset obtained from the MetaQSAR classification system, using a set of common physicochemical descriptors plus stereo-electronic descriptors calculated by either the semiempirical approach (MOPAC) or based on the density functional theory (DFT). The complete list of the molecular descriptors used in the present study is provided in Table S3.

### 2.2. Metaclass^QM^

#### 2.2.1. Classification Models for Classes of Metabolic Reactions

Table 1 reports the predictive performances, obtained by ten-fold cross-validation, for the modelled classes of biotransformations. The full list of metrics, along with the differences in terms of MCC between the models trained on MOPAC and DFT descriptors (ΔMCC), is reported in Table S4. The results revealed that DFT-based models (MCC_mean_ = 0.5) slightly outperformed PM7-based classifiers (MCC_mean_ = 0.45) by about 10% in both phase I and phase II reactions. In more detail, the two sets of descriptors provided comparable performances in terms of MCC for Csp^3^ carbon oxidation, which can be attributed to the high structural diversity and complexity of this class, encompassing a wide range of substrates and metabolic reactions. Moreover, this type of biotransformation is primarily catalyzed by the CYP450 enzyme superfamily, which is known for its ability to metabolize a broad range of chemical structures. Comparable results between DFT- and PM7-based models were also observed for redox reactions of >NH, >NOH, and –N=O class.

The inclusion of DFT-based features notably improves predictions for sulfur (S) redox reactions, methylations, glutathione conjugations, and ester hydrolysis, with sulfur redox reactions showing the highest MCC gain of 25%. For the following classes—oxidation of Csp^3^, oxidation of Csp^2^ and Csp, other hydrolyses, N- and S-glucuronidations and glycosylations, acetylations and acylations—the predictive models obtained using the MOPAC descriptors exhibit quite satisfactory performances (0.4 < MCC < 0.5). Interestingly, the inclusion of DFT-based reactivity parameters slightly improves the discrimination between substrates and non-substrates for other hydrolyses (11%) and N- and S-glucuronidations and glycosylations (10%) classes. The positive role of the DFT-based descriptors can also be appreciated for the following classes—CHOH ↔ C=O → COOH, redox reactions of R_3_N, redox of quinones or analogues, hydrolysis of amides, lactams and peptides, O-glucuronidations and glycosylations, and sulfonations—for which the average increase compared to the PM7-based models exceeds 18%, and is particularly high (>25%) for CHOH ↔ C=O → COOH and redox reactions of R_3_N. Curiously, the DFT-based descriptors proved substantially ineffective for 3 out of 17 classes, namely class 02 (oxidation of Csp^2^ and Csp carbons) and class 26 (CoASH-ligation followed by amino acid conjugations), for which the inclusion of DFT-derived parameters worsened predictive performance by −6.98% and −10%, respectively, compared to MOPAC-based models. This behavior may reflect the limited relevance of DFT-calculated electronic properties for these specific reaction mechanisms. Overall, the performance analysis in terms of mean and standard deviation for the three major reaction classes: redox, hydrolysis, and conjugations, shows similar improvements from DFT-based parameters, with hydrolysis exhibiting the largest average enhancement. In addition, the models’ performance in terms of MCC values confirms the superior predictive ability of DFT features, with a global improvement of approximately 10% for both phase I and phase II metabolism. Moreover, no correlation between the MCC and the dataset size is observed, as shown in Figures S1 and S2 for DFT- and PM7-based models, respectively.

**Figure 1:**
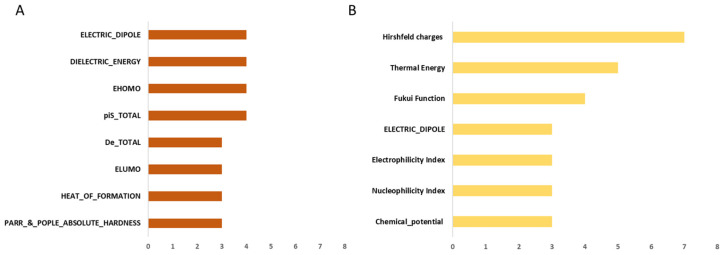
Frequency of the most important MOPAC (**A**) and DFT descriptors (**B**) (i.e., features relevant for at least 3 classes). The color code for the descriptors is the following: dark orange = MOPAC descriptors; light gold = DFT descriptors.

Figure 1 displays the most frequent stereo-electronic descriptors selected by feature selection and used to develop the predictive models. Among the eight most frequently involved PM7-derived stereo-electronic parameters, three are related to the stability of a given molecule (Parr & Pople absolute hardness, heat of formation, and dielectric energy), two are related to the molecular reactivity (piS total and De total), and one is related to polarity (electric dipole moment). The nucleophilicity and electrophilicity are described by the homo and lumo energies. Concerning the most frequent DFT-derived stereo-electronic parameters, four are related to the nucleophilicity and electrophilicity (nucleophilicity index, electrophilicity index, Fukui function, and Hirshfeld charges), while two regard the overall stability/reactivity of a given molecule (chemical potential and thermal energy), and one to polarity (electric dipole moment). The DFT-based models particularly benefit from the inclusion of Hirshfeld charges, as demonstrated in reactions such as redox of sulfur atoms, CHOH ↔ C=O → COOH, ester hydrolysis, methylations, and glutathione conjugations, as further detailed in Table S4. Figure S3 shows the most frequent physico-chemical parameters, including hydrogen bond descriptors (green bars), molecular size parameters (brown bars), and aromaticity/unsaturation parameters (blue bars). Globally, PM7 descriptors were selected as key features for predicting the site of metabolism because they captured aspects of chemical reactivity beyond nucleophilicity and electrophilicity. As an example, PM7 descriptors were able to capture the key interactions with the solvent (dielectric energy), the reactivity of the aromatic system (piS_TOTAL), and the stabilization energy from the attack of an electrophile (De_TOTAL).

#### 2.2.2. Classification Models for Subclasses of Metabolic Reactions

In Table 2, the MCC and AUC values obtained from ten-fold cross-validation of the models generated for all the investigated subclasses are reported. The full list of metrics, including the differences in terms of MCC between MOPAC- and DFT-based classifiers (ΔMCC), is provided in Table S5. A first general observation concerns the comparison between performances at the class and subclass levels. MCC values for subclasses confirm that clustering (i.e., dataset dimensional reduction) improves the overall performance of the predictive models. Specifically, for PM7-based descriptors, clustering into smaller datasets leads to a 20% improvement in MCC for subclasses (MCC__mean_ = 0.54) compared to classes (MCC__mean_ = 0.45). Similarly, for DFT-based models, an improvement of 16% is observed (MCC__mean_ = 0.58 for subclasses vs. 0.50 for classes). The inclusion of DFT-derived stereo-electronic descriptors further increases the average MCC by 8.6% compared to models based on PM7 attributes.

Table 2 highlights the beneficial role of DFT parameters in enhancing the predictive performance for some phase I reactions, such as oxidations of >C=C<, hydrogenations of carbonyls, oxidations of tertiary alkylamines, and oxidations of phenols, for which the MCC improvement exceeds 15%. Regarding hydrolysis reactions, satisfactory models (MCC__mean_ > 0.6) were obtained with either PM7 (MCC__mean_ = 0.69) or DFT descriptors (MCC__mean_ > 0.72). In more detail, highly predictive models (i.e., MCC > 0.7) can be developed for hydrolysis reactions on esters using both PM7 and DFT-based descriptors. Among the three subclasses of ester hydrolysis, only the one involving inorganic acids benefits from the use of DFT attributes, with an MCC improvement greater than 10%. The hydrolysis of anilides and hydrazides is poorly predicted using PM7-based descriptors (MCC < 0.4). However, the inclusion of DFT parameters leads to an improvement of more than 20%, resulting in satisfactory performances (MCC = 0.54). Finally, conjugation reactions show good results (MCC > 0.5) using both PM7 (MCC__mean_ = 0.52) and DFT (MCC__mean_ = 0.57) descriptors, with an improvement of around 8%. The outcomes obtained for the subclasses confirm the difficulty in predicting glucuronidation involving hydroxyl functions, while glucuronidation on carboxylic acids and amines are well modelled, although the enhancing effect of DFT-based descriptors appears to be very modest. Notably, the subdivision into subclasses proves beneficial for modelling glutathione addition-elimination reactions and *O*-sulfonation of phenols, where DFT descriptors improve classification performance by more than 15%. Moreover, no correlation between models’ performances and datasets’ dimensions was detected, as shown in Figures S4 and S5 for PM7- and DFT-based models, respectively. Overall, analysis of mean and standard deviation across subclasses grouped by reaction type (redox, hydrolysis, conjugations) indicates that, regardless of the stereo-electronic descriptors used, reducing dataset size enhances performance by ~10% for oxidation and conjugation and by over 30% for hydrolysis. The impact of DFT descriptors is similar to the previous case of classes, with comparable enhancement.

### 2.3. Metaspot^QM^

#### 2.3.1. Prediction of the Reactive Atoms for the Reaction Classes

The same datasets used to develop MetaClass^QM^ models were subsequently employed to build MetaSpot^QM^ encompassing models for SoM prediction. Table 3 reports the performances in terms of both MCC and AUC yielded by 10-fold cross-validation of the models of the considered classes of metabolic reactions, trained on the two different sets of atomic stereo-electronic descriptors here considered (Table S6): (1) MOPAC atomic descriptors using the PM7 Hamiltonian, (2) DFT descriptors using the B3LYP/6-31G(d) level of theory. The complete list of metrics, along with the differences in MCC between MOPAC and DFT descriptors (ΔMCC), is reported in Table S7.

As displayed in Table 3, satisfactory performances were achieved for most oxidation reactions, ester hydrolyses, and some minor conjugations such as Acetylations/Acylations and CoASH-ligation followed by amino acid conjugations using both MOPAC and DFT descriptors. Focusing on oxidation reactions, only a few classes—oxidation on Csp^3^, CHOH ↔ C=O → COOH, redox reactions of R_3_N, redox of >NH, >NOH, and –N=O, and redox of sulfur atoms—are well predicted with MCC > 0.5 using both sets of stereo-electronic descriptors.

Regarding hydrolysis reactions, satisfactory results (0.4 < MCC < 0.5) are achieved only for reactions involving esters, while other hydrolytic classes show lower performances. Among phase II conjugation reactions, sulfonations, CoASH-ligation followed by amino acid conjugations, and methylations (class 27) are satisfactorily predicted with MCC > 0.5. Overall, the obtained outcomes suggest that the atomic descriptors used to train the models fail to adequately capture reactivity for 8 out of the 17 studied classes (i.e., those with MCC < 0.5). Furthermore, MOPAC and DFT-based models provide similar performances, making it difficult to identify a clear advantage for one descriptor set over the other. Moreover, analyzing all classes grouped by reaction type (redox, hydrolysis, conjugations) based on mean and standard deviation values, the site of metabolism prediction performs better than the prediction of substrate versus non-substrate, as shown by higher mean values. However, it is also accompanied by a higher standard deviation, highlighting that this type of prediction depends more on the reaction type. MOPAC and DFT models yield, on average, similar performance across phase I and phase II metabolism.

**Figure 2:**
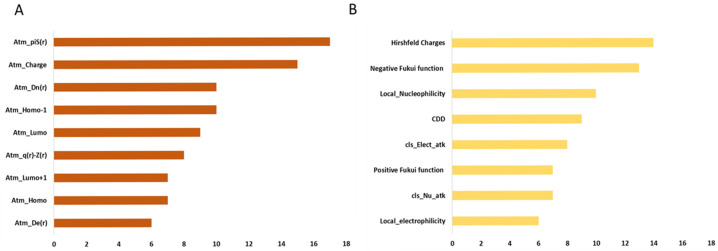
Frequency of the most important MOPAC (**A**) and DFT descriptors (**B**) (i.e., features relevant for at least 3 classes). The color code for the descriptors is the following: dark orange = MOPAC descriptors; light gold = DFT descriptors.

Figure 2 details the frequency of the stereo-electronic descriptors used for models training, calculated as an average across the 17 classes and considering only the features relevant for at least 3 classes. This analysis points out that atomic charges and atomic polarizability (Atm_piS(r)) are the most important among MOPAC descriptors. These attributes encode for the electrophilic/nucleophilic nature of the atoms, along with the reactivity of the π systems. The two descriptors Atm_Dn(r) and Atm_De(r) are associated with stabilization energy from the attack by a nucleophile and electrophile, respectively. Atm_q(r)−Z(r) represents charge density, which is closely related to both nucleophilicity and electrophilicity, but allows intra- and intermolecular charge interactions to be taken into account. Atm_homo/Atm_Lumo, n-HOMO, and n-LUMO are involved in the electron-donating/receiving abilities. Instead, considering the DFT-based features, the most relevant are those describing the reactivity and local nucleophilicity of atoms. In particular, Hirshfeld charges play a major role in identifying the most electrophilic (when positive) and most nucleophilic (when negative) atoms in the molecule. Positive values were used for hydrolysis reactions, and negative values were used for oxidations/conjugations. For the latter reactions, the negative value of the Fukui function is also widely used, confirming the potential role in modeling nucleophilic attacks towards electrophiles, along with the local nucleophilicity and conceptual dual descriptors (CDD), which is a dual feature able to discriminate between electrophilic and nucleophilic sites in a molecule. Globally, PM7 descriptors were selected as key features for predicting the site of metabolism because, compared to DFT descriptors, they provide additional information beyond nucleophilicity and electrophilicity, allowing for a more general description of chemical reactivity.

#### 2.3.2. Prediction of the Reactive Atoms for the Reaction Subclasses

Table 4 reports the site of metabolism prediction for all the subclasses within MetaQSAR. The full list of metrics, along with the differences in terms of MCC for MOPAC and DFT classifiers (ΔMCC), is reported in Table S8. With respect to the previous Metaclass^QM^ approach, here the improvement of the performances related to the dataset dimensional reduction is evident only for some specific subclasses, such as hydrolyses and some phase II conjugation reactions. Overall, satisfactory models were obtained (MCC > 0.5) for some of the phase I metabolic reactions, such as the oxidations involving heteroatoms, as well as all the hydrolysis reactions, with both sets of descriptors. Concerning the phase II metabolic biotransformations, only the reactions involving the glutathione and glucuronidation of carboxylic acids provide satisfactory predictive models. Focusing on phase I reactions, we observed that DFT features allow a better identification of the reactive atoms for the oxidation of unsaturated bond >C=C<, for some oxidations on heteroatoms such as carbonyls, tertiary amines, and phenols, and for all hydrolysis reactions. Instead, phase II biotransformations are better predicted by MOPAC-based models except for the N-glucuronidation of amines and O-sulphonation of phenols, for which enhanced performances are achieved by using DFT-derived attributes.

The reported results suggest that DFT as well as MOPAC parameters can be conveniently used for some specific metabolic reactions, instead of using only one approach for all biotransformations. As previously mentioned, the effect of clusterization in SoM prediction is less evident when compared to Metaclass^QM^. For instance, using PM7 features, the mean MCC slightly decreases from 0.60 (mean value across the classes) to 0.56 (mean value over the subclasses). In contrast, regarding DFT-based models, no differences were detected in terms of mean MCC between classes and subclasses. Overall, analysis of mean and standard deviation across subclasses grouped by reaction type (redox, hydrolysis, conjugations) indicates that, regardless of the stereo-electronic descriptors used, reducing dataset size surprisingly decreases the performance of the redox and conjugation reactions while improving the performance of hydrolysis, considering both MOPAC and DFT features.

## 3. Discussion

Herein, we described the development of Meta^QM^, an ensemble of RF models trained on QM and physicochemical descriptors for metabolism prediction purposes. This work could be considered as an extension of the analysis reported for the MetaClass and MetaSpot tools previously developed by some of us. MetaClass and MetaSpot encompass a set of RF models to predict the occurrence of a given metabolic reaction and SoMs, respectively, and were trained on Kier–Hall topological indices, physicochemical, and PM7-derived descriptors. In MetaQM, which comprises MetaClassQM and MetaSpotQM, two different sets of descriptors were employed. The first included structural, physicochemical, and PM7-derived properties, while, in the second set, PM7 attributes were replaced by DFT-based features, which should, in principle, better describe the stereoelectronic properties of molecules and atoms if compared to semiempirical methods.

Despite a precise comparison of the results obtained here with those reported for MetaClass and MetaSpot being not feasible due to the different criteria by which the non-substrates were chosen in the two studies, some comparative insights can still be drawn.

In more detail, in MetaClass and Metaspot, the negative instances (i.e., non-substrates and non-reactive atoms) were randomly undersampled to obtain balanced classes, while in this work, non-substrates and non-SoMs were selected in order to contain the same functional groups and atom types of those involved in a specific biotransformation. Although this selection strategy makes classification more challenging, it contributes to reducing bias in the results. Interestingly, the use of DFT parameters afforded on average superior performances (mean MCC = 0.49 on classes, mean MCC = 0.54 on subclasses) than MetaClass (mean MCC = 0.46 on classes, mean MCC = 0.48 on subclasses) in the prediction of redox reactions, which are the most heterogeneous among the metabolic biotransformations. This outcome emphasizes that a proper description of molecules’ reactivity can improve the discrimination ability of the models more than the introduction of atom types as encoded by Kier-Hall indices in MetaClass. Comparable results were obtained for hydrolysis, whereas lower performances were observed for conjugation reactions (mean MCC = 0.50 on classes, mean MCC = 0.57 on subclasses) if compared to MetaClass models trained on Kier–Hall indices (mean MCC = 0.59 on classes, mean MCC = 0.66 on subclasses). This can be readily explained by the fact that conjugation reactions primarily involve heteroatoms, and the use of Kier–Hall indices, which encode the presence of specific functional groups, facilitates the discrimination between substrates and randomly selected non-substrates.

Concerning SoM prediction, MetaSpotQM with DFT parameters achieved on average better performances (mean MCC = 0.59 for classes, mean MCC = 0.62 for subclasses) than those obtained in the second round of predictions of MetaSpot (mean MCC = 0.54 for classes, mean MCC = 0.55 for subclasses), in which non-reactive atoms where filtered to have the same atom types of SoMs, as in this work.

In order to provide an unbiased comparison between DFT and semiempirical descriptors for metabolism prediction, we generated models by replacing DFT attributes with MOPAC-derived descriptors. Overall, DFT-based models slightly outperformed PM7-based classifiers for the prediction of the occurrence of a given metabolic reaction, while comparable results were observed for SoMs identification. This outcome suggested that DFT descriptors can provide a finer description of global molecular reactivity with respect to PM7 features, while the two methods furnish a similar depiction of atom reactivity.

Altogether, these observations led to two main considerations. First, the use of atom-type descriptors proved effective only when the two classes include compounds bearing different functional groups, while QM descriptors have a general predictive capability as they account for the intrinsic chemical reactivity. Second, for most of the modelled biotransformation, DFT descriptors afforded models with performances comparable to those trained on PM7 attributes. However, hydrolysis reactions were, on average, better predicted by DFT-based models. This improved performance can be attributed to the more accurate treatment of electron density and frontier orbital energies provided by DFT, which, when combined with physicochemical descriptors, allows a better representation of charge separation, polarization, and hydrogen-bonding effects governing the hydrolytic processes.

In definitive, the models proposed here can be synergistically combined with the previously reported MetaClass and first round MetaSpot classifiers, which include atom type as descriptors, to allow a fast screening of compounds carrying the chemical functionalities susceptible to a specific metabolic reaction. The here proposed QM models can subsequently be used to recognize the true substrates/reactive atoms based on the intrinsic reactivity of the metabolically reactive functions. Such a strategy enables us to reduce the computational costs by applying more demanding QM calculations only to those molecules that surpass the first filter. Furthermore, high-level DFT calculations can be restricted to reaction classes for which DFT-derived descriptors demonstrated superior performance over the MOPAC-based ones, such as hydrolysis.

## 4. Materials and Methods

### 4.1. Preparation of the Datasets

Data extracted from the MetaQSAR database were used to train all models described in this work. MetaQSAR implements a hierarchical classification that subdivides all collected metabolic reactions into 3 major classes (i.e., redox, hydrolysis, and conjugations), 21 classes, and 101 subclasses. Such a classification allows an easy generation of homogeneous datasets that are well suited for predictive analyses. The Metaclass^QM^ approach includes all the classes and subclasses containing at least 50 instances, as previously done by Mazzolari et al. [7]. For each considered class and subclass, the corresponding balanced dataset included all the substrates collected in MetaQSAR with no exception. The non-substrates were collected by applying two filters. The first filter is based on 2D and 3D descriptors to select non-substrates that are reasonably similar to the substrates. Due to its relevance in QM calculations, attention was especially focused on the molecular charge so that the charge average of the substrates is equal to that of the non-substrates. The second filter is based on the analysis of the reactive functional groups so that the selected non-substrates have the same metabolically reactive functions as the substrates. In this way, the classification should be based on a proper evaluation of the intrinsic reactivity of the collected molecules and not on the mere detection of their potentially reactive groups.

The Metaspot^QM^ approach is based on the substrates previously obtained from the Metaclass^QM^ approach. Atoms identified as sites of metabolism, as annotated in MetaQSAR, were labeled as 1, while all other atoms were labeled as 0. A dedicated Python script (version 3.8.0) was developed to efficiently manipulate all input files simultaneously. All datasets were processed to remove duplicates and to extract non-reactive atoms of the same Kier-Hall atom type as the SoMs, in order to highlight and compare the role of stereo-electronic atomic descriptors.

### 4.2. Calculation of Molecular and Atomic Descriptors

MetaClass^QM^ models were built by using two different sets of descriptors. The first set included the structural and physicochemical (PC) descriptors as computed by the VEGA (Drug Design Laboratory, University of Milan, Milan, Italy) suite of programs, plus the stereo-electronic (Elec) descriptors as derived by PM7-based semiempirical calculations performed by means of MOPAC 2016 (Virginia Polytechnic Institute and State University, Blacksburg, VA, USA) [21]. This set is roughly the same already exploited by the first MetaClass [7]. In the second set, the structural and physicochemical descriptors were combined with the DFT-based stereo-electronic parameters computed by Gaussian 16 Revision A.03 (Wallingford, CT, USA) [22]. Further details on the performed DFT calculations and the relative descriptors can be found in [20]. The two sets include a similar number of attributes (44 and 48 for the PM7-based and the DFT-based, respectively), equally subdivided between physicochemical and stereo-electronic attributes, thus allowing a reliable comparison between the stereo-electronic descriptors evaluated using the two levels of theory. Atomic descriptors were computed with MOPAC2016 and Gaussian 16 Revision A.03 using the same level of theory employed for MetaClass^QM^ models.

### 4.3. Generation of the Classification Models for Metaclass^QM^ and MetaSpot^QM^

We built machine learning models to predict whether a compound undergoes a biotransformation and the reactive center based on the MetaQSAR classification system.

For each collected dataset, two models were developed by considering the two sets of descriptors reported in the previous paragraph. A binary classification modeling approach was adopted employing the feature selection strategy and training protocol previously reported [7]. The performance of the developed models was evaluated using the Matthew’s Correlation Coefficient (MCC) and the Area Under the Receiver Operating Characteristic Curve (ROC AUC), due to their robustness and ability to provide a global assessment of model performance [23,24]. These metrics were computed through a 10-fold cross-validation [25] procedure across the extensive set of datasets considered. Further details about the metrics and the validation methods used are reported in the Supplementary Information.

## 5. Conclusions

Based on a highly demanding campaign of DFT calculations, the primary objective of the study was to assess the enhancing role of stereo-electronic descriptors, as computed by applying a high level of theory, for developing classification models for drug metabolism prediction. Specifically, predictive models were built for both classes and subclasses of phase I and phase II metabolic reactions extracted from the MetaQSAR database, comparing the performances obtained using PM7-based and DFT-based descriptors. Two main predictive schemes were considered: (i) Metaclass^QM^ for the discrimination between substrates and non-substrates and (ii) Metaspot^QM^ for SoMs recognition.

In the Metaclass^QM^ approach, models built using DFT-based descriptors showed, on average, a performance improvement of about 10% compared to those based on PM7 descriptors. Notably, some reactions were more accurately predicted (MCC > 0.5) by using the DFT parameters, as observed for redox reactions of sulfur atoms, methylations, conjugations with glutathione, and ester hydrolysis. The largest improvement was achieved for sulfur redox reactions, with an MCC increase of 25% when DFT parameters were used. Concerning the subclass classification system, dataset dimensionality reduction improves the global performances of the predictive models for both sets of molecular descriptors. In detail, the use of PM7 descriptors led to a 20% improvement in MCC for subclasses (MCC_mean_ = 0.54) compared to classes (MCC_mean_ = 0.45). Similarly, DFT-based models showed a 16% improvement in MCC for subclasses (MCC_mean_ = 0.58) compared to classes (MCC_mean_ = 0.50). The inclusion of DFT-based stereoelectronic descriptors led to an improvement in predictive performance of around 9% across the reaction subclasses, compared to models based on MOPAC features.

In the Metaspot^QM^ approach, SoMs were satisfactorily predicted (MCC > 0.5) for most of the redox reactions, hydrolysis of esters, and some minor conjugations, with comparable results between PM7 and DFT descriptor sets. For the subclasses within MetaQSAR, dimensionality reduction improved performance only for hydrolysis and certain conjugation reactions, for which satisfactory models (MCC > 0.5) were obtained.

Overall, this work can be seen as an explorative study concerning the use of two different sets of stereoelectronic descriptors to develop predictive models for metabolism assessment. The reported models could be improved both by enriching the arsenal of the considered descriptors and by including structure-based approaches (such as docking simulations), which can simulate the recognition between the substrate and the involved enzymes. The predictive models could also be optimized by extending and refining the collected metabolic data. Indeed, enriched metabolic data should maximize the chemical space covered by the collected reactions, thus extending the predictive power of the developed models. In addition, enriched metabolic data could be utilized to generate suitable external sets for a more precise validation and tuning of the selected predictive models.

## Figures and Tables

**Table 1:** MCC and AUC values obtained by ten-fold cross-validation for the models developed for the classes of metabolic reactions. (PC indicates physicochemical descriptors, while Elec refers to stereo-electronic features computed by MOPAC or DFT calculations).

Class ID	Description	PC + Elec (MOPAC) MCC	PC + Elec (DFT) MCC	PC + Elec (MOPAC) AUC	PC + Elec (DFT) AUC
01	Oxidation of Csp^3^	0.49	0.49	0.82	0.83
02	Oxidation of Csp^2^ and Csp	0.43	0.4	0.79	0.77
03	CHOH ↔ C=O → COOH	0.36	0.45	0.73	0.79
05	Redox reactions of R_3_N	0.29	0.39	0.69	0.71
06	Redox reactions of >NH, >NOH, and –N=O	0.63	0.62	0.88	0.88
07	Redox of quinones or analogues	0.37	0.41	0.76	0.76
08	Redox of S atoms	0.52	0.65	0.83	0.88
**Main class 1**	**Mean ± Std. dev.**	**0.44 ± 0.11**	**0.49 ± 0.11**	**0.79 ± 0.06**	**0.80 ± 0.06**
11	Hydrolysis of esters, lactones and inorganic esters	0.63	0.7	0.89	0.93
12	Hydrolysis of amides, lactams and peptides	0.3	0.36	0.73	0.74
14	Other hydrolyses	0.44	0.49	0.78	0.82
**Main class 2**	**Mean ± Std. dev.**	**0.46 ± 0.17**	**0.52 ± 0.17**	**0.62 ± 0.37**	**0.64 ± 0.39**
21	O-glucuronidations and glycosylations	0.39	0.45	0.77	0.79
22	N- and S-glucuronidations and glycosylations	0.48	0.53	0.8	0.81
23	Sulfonations	0.32	0.33	0.71	0.69
24	GSH and RSH conjugations	0.65	0.72	0.89	0.91
25	Acetylations and acylations	0.45	0.48	0.77	0.79
26	CoASH-ligation followed by amino acid conjugations	0.5	0.45	0.73	0.74
27	Methylations	0.5	0.54	0.85	0.77
**Main class 3**	**Mean ± Std. dev.**	**0.47 ± 0.10**	**0.50 ± 0.12**	**0.79 ± 0.06**	**0.79 ± 0.07**

**Table 2:** MCC and AUC values obtained by ten-fold cross-validation for the models developed for the subclasses of metabolic reactions. (PC indicates physicochemical descriptors, while Elec refers to stereo-electronic features computed by MOPAC or DFT calculations).

Subclass ID	Description	PC + Elec (MOPAC) MCC	PC + Elec (DFT) MCC	PC + Elec (MOPAC) AUC	PC + Elec (DFT) AUC
01.01	Oxidations of isolated Csp^3^	0.44	0.48	0.80	0.81
01.02	Oxidations of C in α to an unsaturated	0.49	0.50	0.81	0.83
01.03	Oxidations of Csp^3^ carrying an heteroatom	0.47	0.48	0.81	0.83
01.04	Dehydrogenations	0.56	0.57	0.84	0.86
02.01	Oxidations of aryl compounds	0.36	0.39	0.76	0.76
02.02	Oxidations of azarenes	0.46	0.45	0.80	0.80
02.03	Oxidations of >C=C<	0.48	0.56	0.78	0.83
03.02	Hydrogenations of carbonyls	0.45	0.56	0.79	0.77
05.01	Oxidations of tertiary alkylamines	0.37	0.46	0.72	0.77
06.01	Hydroxylations of amines	0.77	0.78	0.92	0.94
07.04	Oxidations of phenols	0.59	0.64	0.87	0.86
08.03	Oxygenations of sulfides	0.58	0.60	0.83	0.87
**Main Subclass 1**	**Mean ± Std. dev.**	**0.50 ± 0.11**	**0.54 ± 0.10**	**0.81 ± 0.05**	**0.83 ± 0.05**
11.01	Hydrolysis of alkyl esters	0.68	0.68	0.90	0.90
11.03	Hydrolysis of anionic and cationic esters	0.85	0.79	0.95	0.96
11.08	Hydrolysis of esters of inorganic acids	0.80	0.88	0.93	0.96
12.02	Hydrolysis of anilides and hydrazides	0.43	0.54	0.77	0.81
**Main Subclass 2**	**Mean ± Std. dev.**	**0.69 ± 0.19**	**0.72 ± 0.15**	**0.89 ± 0.08**	**0.91 ± 0.07**
21.01	O-glucuronidation of alcohols	0.39	0.42	0.74	0.76
21.02	O-glucuronidation of phenols	0.43	0.48	0.80	0.80
21.03	O-glucuronidation of carboxylic acids	0.68	0.70	0.88	0.89
22.01	N-glucuronidation of linear and cyclic amines	0.49	0.52	0.80	0.81
23.01	O-sulfonation of phenols	0.33	0.40	0.75	0.71
24.01	Nucleophilic additions of glutathione	0.81	0.84	0.95	0.95
24.02	Reactions of glutathione addition-elimination	0.52	0.61	0.80	0.85
**Main Subclass 3**	**Mean ± Std. dev.**	**0.52 ± 0.17**	**0.57 ± 0.16**	**0.82 ± 0.07**	**0.82 ± 0.08**

**Table 3:** The performances (as described by accuracy and MCC values) achieved by the classification models for the metabolic reaction classes, considering the two sets of atomic descriptors used, MOPAC and DFT (Elec refers to stereo-electronic features computed by MOPAC or DFT calculations).

Class ID	Description	Elec (MOPAC) MCC	Elec (DFT) MCC	Elec (MOPAC) AUC	Elec (DFT) AUC
01	Oxidation of Csp^3^	0.61	0.56	0.80	0.77
02	Oxidation of Csp^2^ and Csp	0.32	0.38	0.65	0.69
03	CHOH ↔ C=O → COOH	0.63	0.69	0.81	0.84
05	Redox reactions of R_3_N	0.88	0.83	0.94	0.91
06	Redox reactions of >NH, >NOH, and –N=O	0.78	0.70	0.89	0.85
07	Redox of quinones or analogues	0.43	0.45	0.72	0.73
08	Redox of S atoms	0.82	0.71	0.91	0.86
**Main class 1**	**Mean ± Std. dev.**	**0.64 ± 0.21**	**0.62 ± 0.16**	**0.87 ± 0.10**	**0.86 ± 0.08**
11	Hydrolysis of esters, lactones and inorganic esters	0.92	0.92	0.96	0.96
12	Hydrolysis of amides, lactams and peptides	0.42	0.33	0.71	0.67
14	Other hydrolyses	0.42	0.45	0.71	0.72
**Main class 2**	**Mean ± Std. dev.**	**0.59 ± 0.29**	**0.57 ± 0.31**	**0.83 ± 0.13**	**0.80 ± 0.16**
21	O-glucuronidations and glycosylations	0.50	0.41	0.75	0.71
22	N- and S-glucuronidations and glycosylations	0.40	0.51	0.70	0.76
23	Sulfonations	0.49	0.43	0.75	0.72
24	GSH and RSH conjugations	0.53	0.55	0.76	0.77
25	Acetylations and acylations	0.54	0.57	0.77	0.79
26	CoASH-ligation followed by amino acid conjugations	0.77	0.87	0.88	0.94
27	Methylations	0.66	0.67	0.83	0.84
**Main class 3**	**Mean ± Std. dev.**	**0.56 ± 0.12**	**0.57 ± 0.16**	**0.83 ± 0.06**	**0.84 ± 0.06**

**Table 4:** The performances (as described by accuracy and MCC values) achieved by the classification models for the metabolic reaction subclasses, considering the two sets of atomic descriptors used, MOPAC and DFT (Elec refers to stereo-electronic features computed by MOPAC or DFT calculations).

Subclass ID	Description	Elec (MOPAC) MCC	Elec (DFT) MCC	Elec (MOPAC) AUC	Elec (DFT) AUC
01.01	Oxidations of isolated Csp^3^	0.29	0.28	0.69	0.66
01.02	Oxidations of C in α to an unsaturated	0.72	0.62	0.92	0.84
01.03	Oxidations of Csp^3^ carrying an heteroatom	0.54	0.49	0.82	0.80
01.04	Dehydrogenations	0.56	0.45	0.84	0.79
02.01	Oxidations of aryl compounds	0.30	0.32	0.70	0.71
02.02	Oxidations of azarenes	0.68	0.58	0.88	0.86
02.03	Oxidations of >C=C<	0.26	0.46	0.70	0.77
03.02	Hydrogenations of carbonyls	0.57	0.62	0.81	0.85
05.01	Oxidations of tertiary alkylamines	0.74	0.87	0.94	0.93
06.01	Hydroxylations of amines	0.78	0.81	0.96	0.95
07.04	Oxidations of phenols	0.52	0.59	0.79	0.84
08.03	Oxygenations of sulfides	0.78	0.76	0.96	0.94
**Main Subclass 1**	**Mean ± Std. dev.**	**0.56 ± 0.19**	**0.57 ± 0.18**	**0.83 ± 0.10**	**0.83 ± 0.09**
11.01	Hydrolysis of alkyl esters	0.54	0.67	0.83	0.87
11.03	Hydrolysis of anionic and cationic esters	0.78	0.93	0.94	0.99
11.08	Hydrolysis of esters of inorganic acids	0.68	0.86	0.92	0.99
12.02	Hydrolysis of anilides and hydrazides	0.62	0.69	0.85	0.89
**Main Subclass 2**	**Mean ± Std. dev.**	**0.66 ± 0.10**	**0.78 ± 0.13**	**0.89 ± 0.05**	**0.94 ± 0.06**
21.01	O-glucuronidation of alcohols	0.39	0.33	0.70	0.73
21.02	O-glucuronidation of phenols	0.27	0.27	0.69	0.66
21.03	O-glucuronidation of carboxylic acids	0.84	0.87	0.94	0.96
22.01	N-glucuronidation of linear and cyclic amines	0.34	0.48	0.69	0.77
23.01	O-sulfonation of phenols	0.39	0.46	0.77	0.79
24.01	Nucleophilic additions of glutathione	0.67	0.65	0.89	0.87
24.02	Reactions of glutathione addition-elimination	0.60	0.54	0.88	0.80
**Main Subclass 3**	**Mean ± Std. dev.**	**0.50 ± 0.21**	**0.51 ± 0.20**	**0.79 ± 0.11**	**0.80 ± 0.10**

## Data Availability

The original contributions presented in this study are included in the article and Supplementary Materials. Further inquiries can be directed to the corresponding author.

## Supplementary Materials

The following supporting information can be downloaded at https://www.mdpi.com/article/10.3390/ijms262412087/s1. References [23,24,25,26] are cited in Supplementary Materials.
