# Supplementary material for: MetaQM: Exploring the Role of QM Calculations in Drug Metabolism Prediction
Source: Int J Mol Sci. 2025 Dec 16;26(24):12087. doi: 10.3390/ijms262412087 (PMC12732870; doi:10.3390/ijms262412087)
Supplement: Supplementary file 1 [file ijms-26-12087-s001.zip › ijms-4034059-supplementary.pdf]

Meta^{QM}: exploring the role of QM calculations in drug metabolism prediction

Alessio Macorano, Serena Vittorio, Angelica Mazzolari, Alessandro Pedretti, Giulio Vistoli*

[1] Dipartimento di Scienze Farmaceutiche, Università degli Studi di Milano, Via Mangiagalli 25, 20133, Milan, Italy; alessio.macorano@unimi.it (A.M.), serena.vittorio@unimi.it (S.V.), angelica.mazzolari@unimi.it (A.M.), alessandro.pedretti@unimi.it (A.P.), giulio.vistoli@unimi.it (G.V.)

*Correspondence: giulio.vistoli@unimi.it

Table of content:

1. Evaluation metrics binary classification 3

2. Model validation 3

Table S1. Classes of MetaQSAR for which models were developed using physicochemical and stereo-electronic descriptors. 4

Table S2. Subclasses of MetaQSAR for which models were developed using physicochemical and stereo-electronic descriptors. 5

Table S3. List of physicochemical and stereo-electronic molecular descriptors calculated at the semiempirical PM7 and DFT level, with the corresponding description. 6

Table S4. Performance metrics (MCC and AUC values) of Metaclass^{QM} predictive models obtained by 10-fold cross-validation for the 17 considered classes of metabolic reactions 10

Figure S1. Scatter plot of the MCC values of MOPAC-based models within MetaClass^{QM} for all 17 classes in MetaQSAR, plotted against the dataset dimension. 12

Figure S2. Scatter plot of the MCC values of DFT-based models within MetaClass^{QM} for all 17 classes in MetaQSAR, plotted against the dataset dimension. 12

Figure S3. Most frequently selected descriptors (> 7 times). The color code for the descriptors is the following: brown = molecular size; green = H-bonding; blue = aromaticity/unsaturation; yellow = ChiralAtms; orange=lipophilicity. 13

Table S5. Performance metrics (MCC and AUC values) of Metaclass^{QM} predictive models obtained by 10-fold cross-validation for the 23 subclasses of metabolic reactions using the two set of molecular descriptors (physicochemical and MOPAC/DFT). 14

Figure S4. Scatter plot of the MCC values of the MOPAC-based models within MetaSpot^{QM} for all 23 subclasses in MetaQSAR, plotted against the dataset dimension. No correlation is observed between the classification metrics and the dataset's dimension. 16

Figure S5. Scatter plot of the MCC values of the DFT-based models within MetaSpot^{QM}, for all 23 subclasses in MetaQSAR, plotted against the dataset dimension 16

Table S6. List of stereo-electronic atomic descriptors calculated at the semiempirical PM7 and DFT level, with the corresponding description. 17

Table S7. Performance metrics (MCC and AUC values) of MetaSpotQM predictive models for the 17 considered classes of metabolic reactions obtained by ten-fold cross-validation. 18

Table S8. Performance metrics (MCC and AUC values) of MetaSpot^{QM} predictive models for the 23 considered subclasses of metabolic reactions obtained by ten-fold cross-validation. The ΔMCC calculated as the MCC_{DFT} – MCC_{MOPAC} is also reported 20

References..22

1. Evaluation metrics binary classification

The performance of the developed models was evaluated using the Matthew's Correlation Coefficient (MCC) and the Area Under the Receiver Operating Characteristic Curve (ROC AUC), due to their robustness and ability to provide a global assessment of model performance [1] [2]. The MCC is a widely used binary classification metrics which considers all the values in the confusion matrix making it a balanced measure. MCC has values between -1 and +1 where A coefficient of -1 represents no correlation between actual and predicted values, +1 corresponds to a perfect prediction, and 0 indicates a random prediction. The MCC is reported in eq.1:

$$MCC = \frac{TP \cdot TN - FP \cdot FN}{\sqrt{(TP + FP) \cdot (TP + FN) \cdot (TN + FP)(TN + FN)}} \quad (1)$$

The receiver operating characteristic (ROC) curve is a widely used metric for evaluating a model's ability to distinguish between two classes. It is commonly employed in virtual screening campaigns [3]. The ROC curve is produced by computing and plotting the true positive rate (TPR) against the false positive rate (FPR) for the single model classifiers at different thresholds. The area under curve the ROC curve (AUC) represents the probability that, if given a randomly chosen positive and negative sample, the model will rank the positive higher than the negative. The AUC has values between 0 and 1, AUC less than 0.5 indicates that the model performs worse than a random classifier. AUC of 0.5 corresponds to random guessing, and an AUC of 1.0 corresponds to a perfect classifier

2. Model validation

The evaluation metrics previously mentioned were computed using the 10-fold cross-validation methods [4]. The available data are divided into G folds each containing k objects equal to n/G. At each iteration, one fold is used as evaluation set and is therefore excluded from the training process. The model, trained on the remaining n−k objects, is then used to predict the responses of the k excluded objects. Each object is excluded exactly once; after it is reintegrated into the training set, another group of k objects is excluded. In this way, each iii-th object is assigned to a fold G according to the following expression, eq.2:

$$g = mod(i - 1, G) + 1 \quad (2)$$

With mod (c, d) is the modulo operation, c module d

Table S1. Classes of MetaQSAR for which models were developed using physicochemical and stereo-electronic descriptors. Each class is identified by its ID, description, and number of included molecules.

Class ID	*Description*	*Dataset dimension*
01	***Oxidation of Csp^3***	***1486***
02	***Oxidation of Csp^2 and Csp***	***1120***
03	***CHOH ↔ C=O → COOH***	***224***
05	***Redox reactions of R3N***	***238***
06	***Redox reactions of >NH, >NOH, and –N=O***	***310***
07	***Redox of quinones or analogues***	***208***
08	***Redox of S atoms***	***254***
11	***Hydrolysis of esters, lactones and inorganic esters***	***600***
12	***Hydrolysis of amides, lactams and peptides***	***234***
14	***Other hydrolyses***	***190***
21	***O-glucuronidations and glycosylations***	***646***
22	***N- and S- glucuronidations and glycosylations***	***254***
23	***Sulfonations***	***234***
24	***GSH and RSH conjugations***	***338***
25	***Acetylations and acylations***	***138***
26	***CoASH-ligation followed by amino acid conjugations***	***80***
27	***Methylations***	***100***

Table S2. Subclasses of MetaQSAR for which models were developed using physicochemical and stereo-electronic descriptors. Each class is identified by its ID, description, and number of included molecules.

Subclass ID	*Description*	*Dataset dimension*
01.01	***Oxidations of isolated Csp3***	***400***
01.02	***Oxidations of C in α to an unsaturated system***	***486***
01.03	***Oxidations of Csp3 carrying an heteroatom***	***1032***
01.04	***Dehydrogenations***	***164***
02.01	***Oxidations of aryl compounds***	***898***
02.02	***Oxidations of azarenes***	***192***
02.03	***Oxidations of >C=C<***	***108***
03.02	***Hydrogenations of carbonyls***	***132***
05.01	***Oxidations of tertiary alkylamines***	***140***
06.01	***Hydroxylations of amines***	***130***
07.04	***Oxidations of phenols***	***102***
08.03	***Oxygenations of sulfides***	***170***
11.01	***Hydrolysis of alkyl esters***	***194***
11.03	***Hydrolysis of anionic and cationic esters***	***192***
11.08	***Hydrolysis of esters of inorganic acids***	***100***
12.02	***Hydrolysis of anilides and hydrazides***	***120***
21.01	***O-glucuronidation of alcohols***	***170***
21.02	***O-glucuronidation of phenols***	***312***
21.03	***O-glucuronidation of carboxylic acids***	***198***
22.01	***N-glucuronidation of linear and cyclic amines***	***198***
23.01	***O-sulfonation of phenols***	***146***
24.01	***Nucleophilic additions of glutathione***	***178***
24.02	***Reactions of glutathione addition–elimination***	***138***
22.01	***N-glucuronidation of linear and cyclic amines***	***198***
23.01	***O-sulfonation of phenols***	***146***

Table S3. List of physicochemical and stereo-electronic molecular descriptors calculated at the semiempirical PM7 and DFT level, with the corresponding description.

Features (units)	Description
**PM7-based**	
HEAT_OF_FORMATION (kcal/mol)	Enthalpy variation, when one mole of a system is formed from its elements
DIELECTRIC_ENERGY (eV)	Stabilization energy from the interaction of the charges in the solute with the induced charges on the solvent accessible surface plus the electrostatic energy
ELECTRIC_DIPOLE_PM7 (D)	Product between absolute charge and distance between the centre of positive and centre of negative charge
IONIZATION_POTENTIAL_PM7 (eV)	Minimum energy required to eject an electron out of a neutral atom or molecule in its ground state
MOLECULAR_WEIGHT (g/mol)	Mass of a given substance divided by the amount of a substance, defined as mol
COSMO_AREA (Å^2)	Surface of the molecule that can be reached by the by the center of charge of a solvent molecule
COSMO_VOLUME (Å^3)	Volume included in the COSMO surface
CHARGE_ON_SYSTEM	Net charge of the molecule
MULLIKEN_ELECTRONEGATIVITY (eV)	$-\alpha$
PARR_&_POPLE_ABSOLUTE_HARDNESS (eV)	$\eta = ½ (\varepsilon_{homo} - \varepsilon_{lumo})$
SCHUURMANN_MO_SHIFT_ALPHA (eV)	$\alpha = ½ (\varepsilon_{homo} + \varepsilon_{lumo})$, average of the Homo and Lumo energies
EHOMO_PM7 (eV)	Energy of the highest occupied molecular orbital
ELUMO_PM7 (eV)	Energy of the lowest unoccupied molecular orbital
Dn_TOTAL_PM7 (eV^{-1})	Nucleophilic delocalizabilities, Total sums of all $D^N(r)$ (nucleophilic delocalizabilities) for each reactive center (r) of the molecule. It's based on the molecular orbital expansion coefficients, also as a measure of energy stabilization due to nucleophilic attack
De_TOTAL_PM7 (eV^{-1})	Electrophilic delocalizabilities, Total sums of all $D^E(r)$ (electrophilic delocalizabilities) for each reactive centre (r) of a molecule. It’s based on the molecular orbital expansion coefficients, also as a measure for the energy stabilization due to electrophilic attack.
piS_TOTAL (charge^2/energy)	Self-polarizability πS of all atoms
**DFT-based**	

D.E Total_DFT (Hartree^{-1})	Electrophilic delocalizabilities, defined as above, based on the Hirshfeld partition method
D.N Total_DFT (Hartree^{-1})	Nucleophilic delocalizabilities, defined as above, based on the Hirshfeld partition method
VdW_volume (Å^3)	Molecular volume based on vdW radii
Ehomo_DFT (eV)	Energy of the highest occupied molecular orbital
Elumo_DFT (eV)	Energy of the lowest unoccupied molecular orbital
Gap (eV)	Difference between homo and lumo energy
dipole_DFT (D)	Product between absolute charge and distance between the centre of positive and centre of negative charge
Chemical_potential (eV)	Energy change of a system with respect to electron number at a fixed external potential
Electron_affinity_DFT (eV)	Energy change when an electron is added to a neutral molecule
Mulliken_electronegativity_DFT (eV)	Measure of resistance to the loss of electron density
Ionization_potential_DFT (eV)	Minimum energy required to eject an electron out of a neutral atom or molecule in its ground state
Hardness_DFT (eV)	Change of the electronic chemical potential respect to the electron number at a fixed external potential
Softness (eV)	Inverse of the chemical hardness
Electrophilicity_index (eV)	Electrophilicity of a molecule, a measure of the energy stabilization of a molecule when it acquires an additional amount of electron density from the environment
Nucleophilicity_index (eV)	Nucleophilicity of a molecule, expressed as the energy difference between homo of the nucleophile and homo of tetracyanoethylene
ThermalEnergy (kcal/mol)	Sum of electronic and thermal energies
Electronic_ZPE (kcal/mol)	Sum of electronic and zero-point vibrational energy
Enthalpy (kcal/mol)	Sum of electronic and thermal enthalpies
Gibbs_energy (kcal/mol)	Sum of electronic and thermal free energies
Hirshfeld_positive_charges (e)	Most positive value of atomic Hirshfeld charge
Fukui_positive (e)	Most positive value of positive Fukui function (Nucleophilic attack)
CDD_positive (e)	Most positive value of CDD (Difference between positive and negative Fukui function)
Hirshfeld_negative_charges (e)	Most negative value of atomic Hirshfeld charge

Fukui_negative (e)	Most positive value of the negative Fukui function (Electrophilic attack)
CDD_negative (e)	Most negative value of CDD (Difference between positive and negative Fukui function)
**Physicochemical descriptors**	
Angles	Number of angles
Atoms	Number of atoms
Bonds	Number of bonds
Charge (e)	Total charge
ChiralAtms	Number of chiral atoms
Dipole	Product between absolute charge and distance between centre of positive and centre of negative charge
EzBnds	Number of bonds with E/Z geometry
FlexTorsions	Number of rotable bonds
Gyrrad (Å)	Radius of gyration. Measure of the distribution of atoms in a molecular structure with respect to either its center of mass or a given axis of rotation
HbAcc	Number of H-bond acceptor atoms
HbDon	Number of H-bond donor atoms
HeavyAtoms	Number of heavy atoms
Impropers	Number of improper angles
Lipole	Lipophilicity moment, measure of the lipophilic distribution in a 3D space
Mass (Da)	Molecular weight
MassMI	Monoisotopic mass
Ovality	Ratio Area/Volume, measure of how close the shape of a molecule is to a sphere or a cigar shape
Psa (Å^2)	Polar surface area, is defined as the amount of molecular surface arising from polar atoms (nitrogen and oxygen atoms together with their attached hydrogen atoms
Rings	Number of rings in the molecule
Sas (Å^2)	Solvent accessible surface
Sav (Å^3)	Solvent accessible volume
Sdiam (Å)	Surface diameter, diameter of the equivalent sphere with the same surface of the molecule
Surface (Å^2)	Molecular surface

Torsions	Number of torsion angles
Vdiam (Å)	Volume diameter, diameter of the equivalent sphere with the same volume of the molecule
VirtualLogP	Lipophilicity measure
Volume (Å^3)	Molecular volume

Table S4. Performance metrics (MCC and AUC values) of Metaclass^{QM} predictive models obtained by 10-fold cross-validation for the 17 considered classes of metabolic reactions using the two set of molecular descriptors (physicochemical and MOPAC / DFT). The ΔMCC calculated as the MCC_{DFT} – MCC_{MOPAC} is also reported.

Class ID	Description	Dataset dimension	PC + Elec (MOPAC) MCC	PC + Elec (DFT) MCC	PC + Elec (MOPAC) AUC	PC + Elec (DFT) AUC	ΔMCC (DFT-MOPAC)
01	**Oxidation of Csp^3**	1486	0.49	0.49	0.82	0.83	0
02	**Oxidation of Csp^2 and Csp**	1120	0.43	0.4	0.79	0.77	-0.03
03	**CHOH ↔ C=O → COOH**	224	0.36	0.45	0.73	0.79	0.09
05	**Redox reactions of R3N**	238	0.29	0.39	0.69	0.71	0.1
06	**Redox reactions of >NH, >NOH, and –N=O**	310	0.63	0.62	0.88	0.88	-0.01
07	**Redox of quinones or analogues**	208	0.37	0.41	0.76	0.76	0.04
08	**Redox of S atoms**	254	0.52	0.65	0.83	0.88	0.13
11	**Hydrolysis of esters, lactones and inorganic esters**	600	0.63	0.7	0.89	0.93	0.07
12	**Hydrolysis of amides, lactams and peptides**	234	0.3	0.36	0.73	0.74	0.06
14	**Other hydrolyses**	190	0.44	0.49	0.78	0.82	0.05
21	**O-glucuronidations and glycosylations**	646	0.39	0.45	0.77	0.79	0.06
22	**N- and S-glucuronidations and glycosylations**	254	0.48	0.53	0.8	0.81	0.05
23	**Sulfonations**	234	0.32	0.33	0.71	0.69	0.01

24	**GSH and RSH conjugations**	338	0.65	0.72	0.89	0.91	0.07
25	**Acetylations and acylations**	138	0.45	0.48	0.77	0.79	0.03
26	**CoASH-ligation followed by amino acid conjugations**	80	0.5	0.45	0.73	0.74	-0.05
27	**Methylations**	100	0.5	0.54	0.85	0.77	0.04

Figure S1. Scatter plot of the MCC values of MOPAC-based models within MetaClass^{QM} for all 17 classes in MetaQSAR, plotted against the dataset dimension. No correlation is observed between the classification metrics and the dataset's dimension.

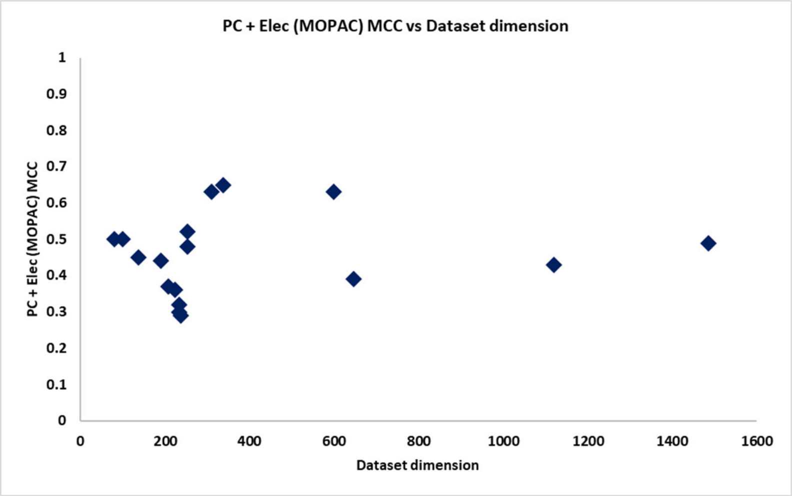

Figure S2. Scatter plot of the MCC values of DFT-based models within MetaClass^{QM} for all 17 classes in MetaQSAR, plotted against the dataset dimension. No correlation is observed between the classification metrics and the dataset's dimension.

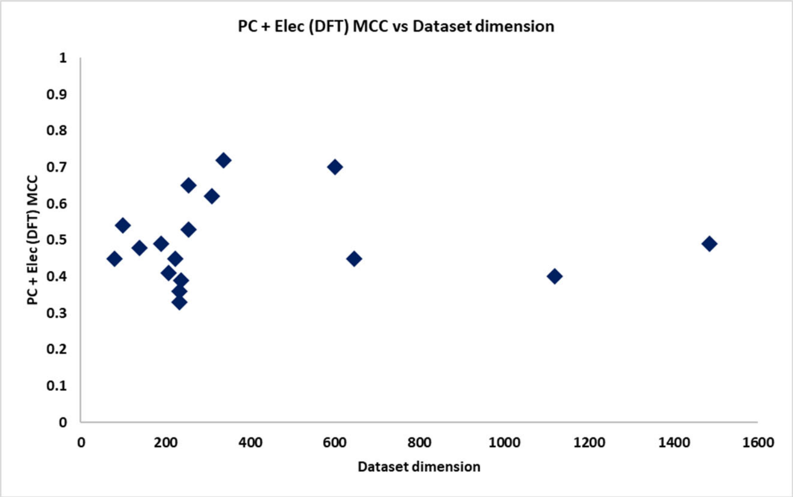

Figure S3. Most frequently selected descriptors (> 7 times). The color code for the descriptors is the following: brown = molecular size; green = H-bonding; blue = aromaticity/unsaturation; yellow = ChiralAtms; orange=lipophilicity.

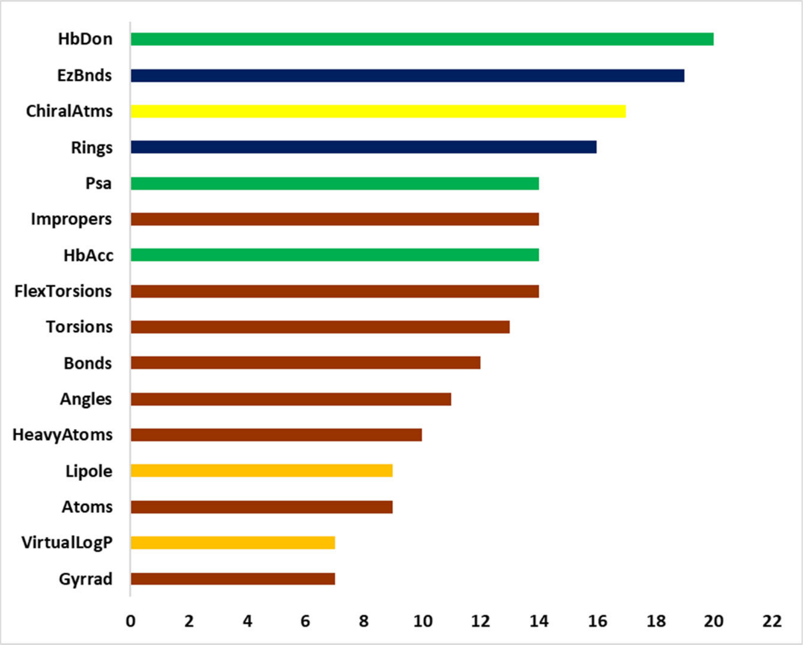

Table S5. Performance metrics (MCC and AUC values) of Metaclass^{QM} predictive models obtained by 10-fold cross-validation for the 23 subclasses of metabolic reactions using the two set of molecular descriptors (physicochemical and MOPAC/DFT). The ΔMCC calculated as the $MCC_{DFT} - MCC_{MOPAC}$ is also reported.

Subclass ID	Description	Dataset dimension	PC + Elec (MOPAC) MCC	PC + Elec (DFT) MCC	PC + Elec (MOPAC) AUC	PC + Elec (DFT) AUC	ΔMCC (DFT-MOPAC)
01.01	**Oxidations of isolated Csp³**	400	0.44	0.48	0.80	0.81	0.04
01.02	**Oxidations of C in α to an unsaturated system**	486	0.49	0.50	0.81	0.83	0.01
01.03	**Oxidations of Csp³ carrying an heteroatom**	1032	0.47	0.48	0.81	0.83	0.01
01.04	**Dehydrogenations**	164	0.56	0.57	0.84	0.86	0.01
02.01	**Oxidations of aryl compounds**	898	0.36	0.39	0.76	0.76	0.03
02.02	**Oxidations of azarenes**	192	0.46	0.45	0.80	0.80	-0.01
02.03	**Oxidations of >C=C<**	108	0.48	0.56	0.78	0.83	0.08
03.02	**Hydrogenations of carbonyls**	132	0.45	0.56	0.79	0.77	0.11
05.01	**Oxidations of tertiary alkylamines**	140	0.37	0.46	0.72	0.77	0.09
06.01	**Hydroxylations of amines**	130	0.77	0.78	0.92	0.94	0.01
07.04	**Oxidations of phenols**	102	0.59	0.64	0.87	0.86	0.05
08.03	**Oxygenations of sulfides**	170	0.58	0.60	0.83	0.87	0.02
11.01	**Hydrolysis of alkyl esters**	194	0.68	0.68	0.90	0.90	0.00

11.03	**Hydrolysis of anionic and cationic esters**	192	0.85	0.79	0.95	0.96	-0.06
11.08	**Hydrolysis of esters of inorganic acids**	100	0.80	0.88	0.93	0.96	0.08
12.02	**Hydrolysis of anilides and hydrazides**	120	0.43	0.54	0.77	0.81	0.11
21.01	**O-glucuronidation of alcohols**	170	0.39	0.42	0.74	0.76	0.03
21.02	**O-glucuronidation of phenols**	312	0.43	0.48	0.80	0.80	0.05
21.03	**O-glucuronidation of carboxylic acids**	198	0.68	0.70	0.88	0.89	0.02
22.01	**N-glucuronidation of linear and cyclic amines**	198	0.49	0.52	0.80	0.81	0.03
23.01	**O-sulfonation of phenols**	146	0.33	0.40	0.75	0.71	0.07
24.01	**Nucleophilic additions of glutathione**	178	0.81	0.84	0.95	0.95	0.03
24.02	**Reactions of glutathione addition−elimination**	138	0.52	0.61	0.80	0.85	0.09

Figure S4. Scatter plot of the MCC values of the MOPAC-based models within MetaSpot^{QM} for all 23 subclasses in MetaQSAR, plotted against the dataset dimension. No correlation is observed between the classification metrics and the dataset's dimension.

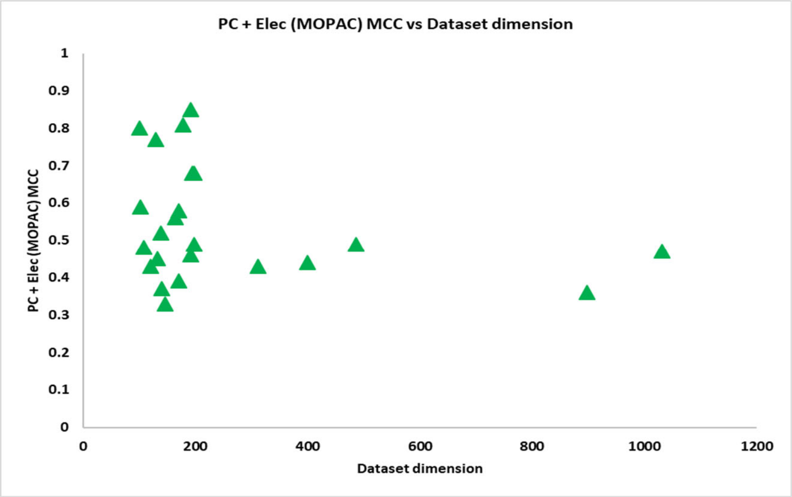

Figure S5. Scatter plot of the MCC values of the DFT-based models within MetaSpot^{QM}, for all 23 subclasses in MetaQSAR, plotted against the dataset dimension. No correlation is observed between the classification metrics and the dataset's dimension.

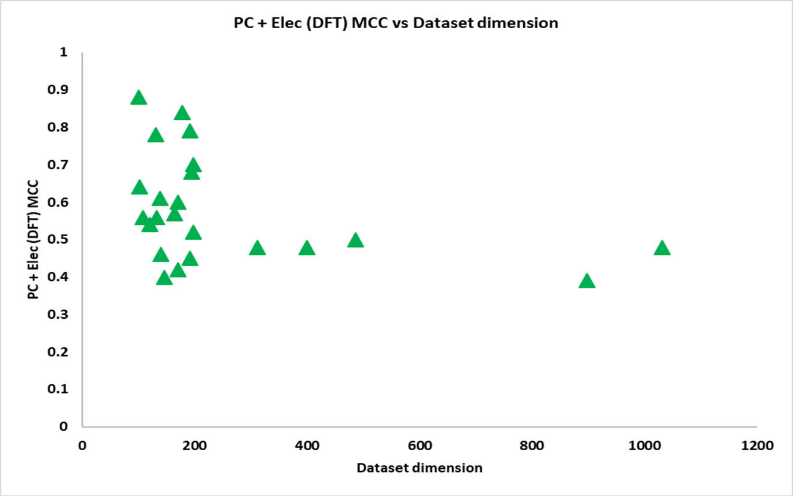

Table S6. List of stereo-electronic atomic descriptors calculated at the semiempirical PM7 and DFT level, with the corresponding description.

Atomic Features (units)	Description
**PM7-based**	
Atm_Dn(r) (eV^{-1})	Atomic nucleophilic delocalizabilities
Atm_De(r) (eV^{-1})	Atomic electrophilic delocalizabilities
Atm_q(r)-Z(r) (e)	Atomic charge density
Atm_piS(r) ($charge^2$/energy)	Atomic self-polarizability πS
Atm_Homo-1 (%)	Atomic % of the electron density of the Homo-1 orbital
Atm_Homo (%)	Atomic % of the electron density of the Homo orbital
Atm_Lumo (%)	Atomic % of the electron density of the Lumo orbital
Atm_Lumo+1(%)	Atomic % of the electron density of the Homo-1 orbital
**DFT-based**	
Hirshfeld_charges (e)	Hirshfeld charges, obtained by HPA
Negative Fukui function (e)	Fukui function for nucleophilic reactive sites
Positive Fukui function (e)	Fukui function for electrophilic reactive sites
Conceptual_dual_descriptor (e)	Atomic value of CDD
Local_electrophilicity (e)	Local condensed electrophilicity
Local_Nucleophilicity(e)	Local condensed nucleophilicity
cls_Nu_atk (e)	Condensed local softness for electrophilic attack
cls_Elect_atk (e)	Condensed local softness for nucleophilic attack

Table S7. Performance metrics (MCC and AUC values) of MetaSpotQM predictive models for the 17 considered classes of metabolic reactions obtained by ten-fold cross-validation. The ΔMCC calculated as the $MCC_{DFT} - MCC_{MOPAC}$ is also reported.

Class ID	Description	Elec (MOPAC) MCC	Elec (DFT) MCC	Elec (MOPAC) AUC	Elec (DFT) AUC	ΔMCC (DFT-MOPAC)
01	**Oxidation of Csp^3**	0.61	0.56	0.85	0.83	-0.05
02	**Oxidation of Csp^2 and Csp**	0.32	0.38	0.71	0.74	0.06
03	**CHOH ↔ C=O → COOH**	0.63	0.69	0.89	0.92	0.06
05	**Redox reactions of R3N**	0.88	0.83	0.98	0.95	-0.05
06	**Redox reactions of >NH, >NOH, and –N=O**	0.78	0.70	0.93	0.91	-0.08
07	**Redox of quinones or analogues**	0.43	0.45	0.79	0.77	0.02
08	**Redox of S atoms**	0.82	0.71	0.96	0.92	-0.11
11	**Hydrolysis of esters, lactones and inorganic esters**	0.92	0.92	0.98	0.98	0.00
12	**Hydrolysis of amides, lactams and peptides**	0.42	0.33	0.76	0.68	-0.09
14	**Other hydrolyses**	0.42	0.45	0.75	0.74	0.03
21	**O-glucuronidations and glycosylations**	0.50	0.41	0.80	0.75	-0.09
22	**N- and S-glucuronidations and glycosylations**	0.40	0.51	0.75	0.82	0.11
23	**Sulfonations**	0.49	0.43	0.80	0.80	-0.06

24	**GSH and RSH conjugations**	0.53	0.55	0.81	0.83	0.02
25	**Acetylations and acylations**	0.54	0.57	0.84	0.88	0.03
26	**CoASH-ligation followed by amino acid conjugations**	0.77	0.87	0.92	0.95	0.10
27	**Methylations**	0.66	0.67	0.88	0.84	0.01

Table S8. Performance metrics (MCC and AUC values) of MetaSpot^{QM} predictive models for the 23 considered subclasses of metabolic reactions obtained by ten-fold cross-validation. The ΔMCC calculated as the $MCC_{DFT} - MCC_{MOPAC}$ is also reported.

Subclass ID	Description	Elec (MOPAC) MCC	Elec (DFT) MCC	Elec (MOPAC) AUC	Elec (DFT) AUC	ΔMCC (DFT-MOPAC)
01.01	**Oxidations of isolated Csp^3**	0.29	0.28	0.69	0.66	-0.01
01.02	**Oxidations of C in α to an unsaturated system**	0.72	0.62	0.92	0.84	-0.10
01.03	**Oxidations of Csp^3 carrying an heteroatom**	0.54	0.49	0.82	0.80	-0.05
01.04	**Dehydrogenations**	0.56	0.45	0.84	0.79	-0.11
02.01	**Oxidations of aryl compounds**	0.30	0.32	0.70	0.71	0.02
02.02	**Oxidations of azarenes**	0.68	0.58	0.88	0.86	-0.10
02.03	**Oxidations of >C=C<**	0.26	0.46	0.70	0.77	0.20
03.02	**Hydrogenations of carbonyls**	0.57	0.62	0.81	0.85	0.05
05.01	**Oxidations of tertiary alkylamines**	0.74	0.87	0.94	0.93	0.13
06.01	**Hydroxylations of amines**	0.78	0.81	0.96	0.95	0.03
07.04	**Oxidations of phenols**	0.52	0.59	0.79	0.84	0.07
08.03	**Oxygenations of sulfides**	0.78	0.76	0.96	0.94	-0.02
11.01	**Hydrolysis of alkyl esters**	0.54	0.67	0.83	0.87	0.13

11.03	**Hydrolysis of anionic and cationic esters**	0.78	0.93	0.94	0.99	0.15
11.08	**Hydrolysis of esters of inorganic acids**	0.68	0.86	0.92	0.99	0.18
12.02	**Hydrolysis of anilides and hydrazides**	0.62	0.69	0.85	0.89	0.07
21.01	**O-glucuronidation of alcohols**	0.39	0.33	0.70	0.73	-0.06
21.02	**O-glucuronidation of phenols**	0.27	0.27	0.69	0.66	0.00
21.03	**O-glucuronidation of carboxylic acids**	0.84	0.87	0.94	0.96	0.02
22.01	**N-glucuronidation of linear and cyclic amines**	0.34	0.48	0.69	0.77	0.14
23.01	**O-sulfonation of phenols**	0.39	0.46	0.77	0.79	0.07
24.01	**Nucleophilic additions of glutathione**	0.67	0.65	0.89	0.87	-0.02
24.02	**Reactions of glutathione addition−elimination**	0.60	0.54	0.88	0.80	-0.06

References

1. Chicco, D.; Jurman, G. The Advantages of the Matthews Correlation Coefficient (MCC) over F1 Score and Accuracy in Binary Classification Evaluation. *BMC Genomics* **2020**, *21*, 6, doi:10.1186/s12864-019-6413-7.

2. Osborn, J. Fundamentals of Biostatistics. Bernard Rosner, Third Edition, PWS-Kent, Boston, 1990. No. of Pages: Xv + 655. Price: £14.95 (International Student Edition). *Stat Med* **1991**, *10*, 1783–1783, doi:10.1002/sim.4780101115.

3. Empereur-mot, C.; Guillemain, H.; Latouche, A.; Zagury, J.-F.; Viallon, V.; Montes, M. Predictiveness Curves in Virtual Screening. *J Cheminform* **2015**, *7*, 52, doi:10.1186/s13321-015-0100-8.

4. Forina, M.; Drava, G.; Boggia, R.; Lanteri, S.; Conti, P. Validation Procedures in Near-Infrared Spectrometry. *Anal Chim Acta* **1994**, *295*, 109–118, doi:10.1016/0003-2670(94)80340-4.
